# Two New Triterpene Glycosides with Monomethyl Malonate Groups from the Rhizome of *Cimifuga foetida* L. 

**DOI:** 10.3390/molecules16075701

**Published:** 2011-07-05

**Authors:** Li-Rong Sun, Jian Yan, Lin Zhou, Zhong-Rong Li, Ming-Hua Qiu

**Affiliations:** 1 State Key Laboratory of Phytochemistry and Plant Resources in West China, Kunming Institute of Botany, the Chinese Academy of Sciences, Kunming 650204, China; 2 Department of Neurobiology, Southern Medical University, Guangzhou 510515, China; 3 Key Laboratory of Plant Resources Conservation and Sustainable Utilization, South China Botanical Garden, Chinese Academy of Sciences, Guangzhou 510650, China; Email: yanjian@scbg.ac.cn

**Keywords:** *Cimicifuga foetida*, cycloartane triterpenoid, cimiaceroside, cimifoside

## Abstract

Two new 9,19-cycloartane triterpene glycosides **1**-**2**, together with four known compounds—26-deoxyactein (**3**), actein (**4**), 7,8-didehydro-26-deoxyactein (**5**) and cimiaceroside B (**6**)—were isolated from the rhizome of *Cimicifuga foetida*. The new triterpene glycosides were identified as 23-O-methyl-24-deoxy-2'-O-(3''-methylmalonyl)-cimiaceroside B (**1**) and 2'-O-(3''-methylmalonyl)actein (**2**) based on analysis of their spectral data and chemical reactions.

## 1. Introduction

**Figure 1:**
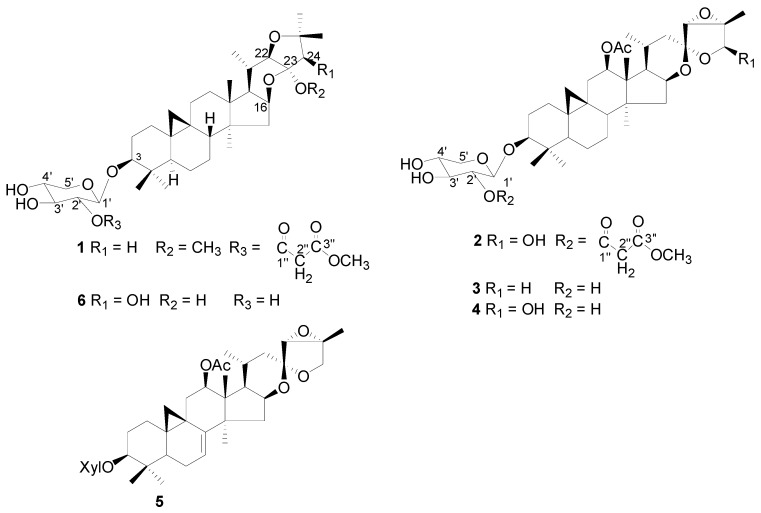
Chemical structures of triterpenoid glycoside from the *C. foetida.*

Cimicifuga species are popular Chinese Traditional Medicine, and they are also widely used as a herbal dietary supplement for the relief of symptoms related to menopause [1,2] with a clinical history going back over forty years [3] in the United States and the European Union. Previous investigations revealed that triterpene glycosides were the main active components and could be used as marker compounds to standardize the extracts [4]. In our continuing to search on *C. foetida* from different geographic regions, we have separated more than 40 triterpene glycosides [5,6,7,8,9,10,11]. In the present investigation on *C. foetida *collected in Lijiang county, Yunnan Province, two new glycosides **1**-**2**, besides the known compounds 26-deoxyactein (**3**) [12], actein (**4**) [13], 7,8-didehydro-26-deoxyactein (**5**) [14] and cimiaceroside B (**6**) [15] were isolated from it (Figure 1). This report describes the isolation and structure elucidation of the new compounds.

## 2. Results and Discussion

**Figure 2:**
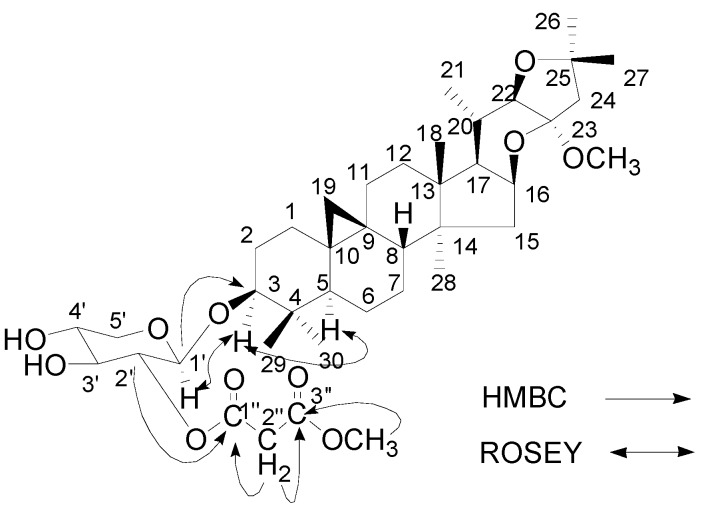
The key HMBC and ROSEY correlations of **1**.

Compound **1** was isolated as a white powder. Its molecular formula was determined as C_40_H_62_O_11_ deduced from the negative HRFABMS (*m/z *717.3146 [M-H]^-^, calcd. 717.3158 for C_40_H_61_O_11_) and the ^1^H- and ^13^C-NMR spectra. The IR spectrum of **1** showed absorptions at 3,454, 1,728 and 1,211 cm^−1^ due to hydroxyl and carbonyl groups. The overall physical properties and NMR spectral profile suggested a cycloartane type of triterpene glycoside, a characteristic and distinguishable chemical marker of *Cimicifuga *plants [12]. The characteristic cyclopropane methylene signals at δ_H_ 0.13 and 0.39 (each 1H, d, *J* = 2.9 Hz); six tertiary methyl groups at δ_H_ 1.14 (Me-18), 1.51 (Me-26), 1.61 (Me-27), 0.83 (Me-28), 1.30 (Me-29), 0.97 (Me-30) (each 3H, s), and one secondary methyl group at δ_H_ 1.15 (3H, d, *J *= 6.4 Hz, Me-21); one methylene signal at δ_H_ 3.78 (2H, s, H-2''); and two methoxyl groups at δ_H_ 3.36 (3H, s) and δ_H_ 3.65 (3H, s); and an anomeric proton at δ_H_ 4.83（d, *J* = 6.4 Hz) were observed in the ^1^H-NMR spectrum (see Table 1). The ^13^C-NMR spectrum of **1 **displayed 40 carbons, of which 30 carbons were ascribable to the triterpene aglycone, five carbons to a pentose residue at δ_C_ 104.2 (C-1'), 76.8 (C-2'), 76.6 (C-3'), 71.3 (C-4'), 67.1 (C-5'), four carbons to one monomethyl malonate group at δ_C_ 166.5 (C-1''), 42.0 (C-2''), 167.2 (C-3'') and 52.3 (3''-OCH_3_), and one carbons to one methoxyl at δ_C_ 49.6 (24-OCH_3_) (see Table 1). The NMR spectroscopic data of **1 **showed great resemblance with those of 2'-O-malonylcimiaceroside B [16], except for the changes of C-3'', C-23, C-24, and the location of the monomethyl malonate group was also assigned to C-2' of the xylose unit in the following elucidation. First, all δ_C_ and δ_H_ of **1** were assigned by a detailed analysis of the HSQC spectrum. Then, in the HMBC spectrum, an informative correlation was observed between H-1' of the anomeric signal at δ_H_ 4.89 and a CH signal at δ_C_ 88.5 (C-3), suggesting that the sugar moiety was linked to C-3. After acid hydrolysis, only xylose was identified in the aqueous fraction by TLC comparison with an authentic sample, indicating the sugar unit in **1** was xylose. Moreover, the proton coupling constants of H-1' (*J* = 6.4 Hz) suggested **1** had a *β*-D-xylopyranoside moiety, which was further supported by an obvious correlation between H-1' (δ_H_ 4.89) of the xylose unit and H-3 (δ_H_ 3.60) of the aglycon in the ROESY spectrum (Figure 2). A significant ROESY correlation between H-3 and H-5 suggested a *β*-orientation of the substituent group at C-3. Furthermore, the location of the monomethyl malonate group could be unambiguously assigned to C-2' of the xylose unit by HMBC, as a correlation was observed between H-2' (δ_H_ 5.54, t, *J *= 6.9 Hz) and the carbonyl signal at δ_C_ 166.5. In the HMBC, two conspicuous correlations could also be observed between the methoxyl (δ_H_ 3.65) and the carbonyl (C-3'', δ_C_ 167.2), and between the methoxyl (δ_H_ 3.36) and the hemiacetal (C-23, δ_C_ 109.7), which suggested that the one methoxyl located the malonyl group to form a monomethyl malonate group, and another methoxyl was linked to C-23. There was a notable difference at C-24 between compound **1 **and 2'-O-malonylcimiaceroside B, namely a methine signal at C-24 in 2'-O-malonylcimiaceroside B, while a methylene signal was observed at C-24 (δ_C_ 30.0, t) in compound **1**, which was confirmed by HMBC correlations from δ_H_ 1.54 (m, H-24) to δ_C_ 109.7 (s, C-23) and to 83.4 (s, C-25). Thus, compound **1** was determined to be 23-*O*-methyl-24-deoxy-2'-*O*-(3''-methylmalonyl)-cimiaceroside B, named cimiaceroside E.

**Table 1:** The ^1^H and ^13^C-NMR data of **1 **and **2** in C_5_D_5_N (δin ppm).

No	1 ^a^		2 ^b^		No	1 ^a^		2 ^b^	
	^13^C	^1^H	^13^C	^1^H		^13^C	^1^H	^13^C	^1^H
1	32.0 t	1.20 m; 1.52 m	31.8 t	1.15 m; 1.52 m	20	34.3 d	2.15 m	26.0 d	1.79 m
2	29.8 t	1.85 m; 2.20 m	29.7 t	1.88 m; 2.28 m	21	17.5 q	1.15 d 6.4	21.0 q	0.94 d 6.3
3	88.5 d	3.39 d 3.5	88.2 d	3.31 dd 4.5, 11.5	22	86.3 d	3.67 m	37.6 t	1.65 m; 2.20 m
4	41.0 s	/	40.9 s	/	23	109.7 s	/	105.8 s	/
5	47.3 d	1.33 m	46.9 d	1.26 m	24	30.0 t	1.54 m	63.5 d	3.90 s
6	20.9 t	0.73 m; 1.31 m	20.1 t	0.65 m; 1.45 m	25	83.4 s	/	65.6 s	/
7	26.1 t	1.61 m	25.7 t	0.95 m; 1.30 m	26	27.1 q	1.51 s	98.4 d	5.70 s
8	47.5 d	1.49 m	45.8 d	1.52 m	27	24.6 q	1.61 s	13.1 q	1.74 s
9	19.7 s	/	20.4 s	/	28	19.7 q	0.83 s	19.5 q	0.75 s
10	26.4 s	/	26.7 s	/	29	25.6 q	1.30 s	25.6 q	1.08 s
11	26.5 t	1.79 m	36.7 t	1.16 m; 2.68 dd 8.3, 15.9	30	15.2 q	0.97 s	15.1 q	0.91 s
12	33.4 d	1.50 m	77.1 d	5.05 dd 3.7, 8.5	1'	104.2 d	4.83 d 7.4	104.1 d	4.79 d 7.8
13	46.9 s	/	48.7 s	/	2'	76.8 d	5.54 t 6.9	76.7 d	5.50 t 8.4
14	45.3 s	/	47.8 s	/	3'	76.6 d	4.15 m	76.1 d	4.11 m
15	42.8 t	1.58 m; 1.87 m	43.6 t	1.89 dd 8.1, 12.6; 1.71 m	4'	71.3 d	4.18 m	71.3 d	4.15 m
16	72.5 d	4.40 dd 6.0, 12.5	73.0 d	4.58 dd 7.1, 14.2	5'	67.1 t	3.64 m; 4.29 dd 3.5, 8.7	67.1 t	3.60 m; 4.25 dd 7.1, 11.9
17	51.6 d	1.50 m	56.4 d	1.77 m	1''	166.5 s	/	166.5 s	/
18	20.6 q	1.14 s	13.6 q	1.33 s	2''	42.0 t	3.78 s	42.0 t	3.74 s
19	30.2 t	0.13 d 2.9; 0.39 d 2.9	29.6 t	0.17 d 4.0; 0.50 d 4.0	3''	167.2 s	/	167.2 s	**/**

Note: ^a^ 23-OCH_3_ (δ_C_ 49.6, q; δ_H_ 3.36, s); 3''-CH_3_O (δ_C_ 52.3, q; δ_H_ 3.65, s); ^b^ 12-COCH_3_ (δ_C_ 170.6, s), 12-COCH_3_ (δ_C_ 21.7, q; δ_H_ 2.12, s); 3''-CH_3_O (δ_C_ 52.3, q; δ_H_ 3.63, s).

**Figure 3:**
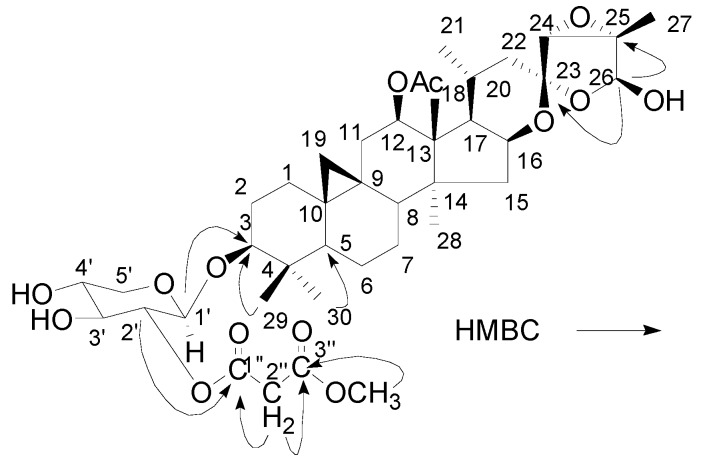
The key HMBC correlations of **2**.

Compound **2** was isolated as a white powder. The negative HR-FAB-MS of **2** showed a quasimolecular ion at *m/z *775.4365, corresponding to the molecular formula of C_41_H_60_O_14_. The IR spectrum of **2** showed absorptions at 3,448, 2,500–3,000, 1,737 and 1,244 cm^−1^ due to hydroxyl and carbonyl groups. Its ^1^H-NMR spectrum (Table 1) exhibited characteristic cyclopropane methylene signals at δ_H_ 0.17 and 0.50 (each 1H, d, *J *= 4.0 Hz); seven methyls at δ_H_ 1.33 (3H, s, Me-18), 0.94 (3H, d, *J *= 6.3 Hz, Me-21), 5.70 (3H, s, Me-26), 1.70 (3H, s, Me-27), 0.75 (3H, s, Me-28), 1.08 (3H, s, Me-29) and 0.91 (3H, s, Me-30); Additionally, other signals for an anomeric proton at δ_H_ 4.79 (1H, d, *J *= 7.8 Hz, H-1'), for an acetyl group at δ_H_ 2.12 (3H, s), and for a monomethyl malonate group at δ_H_ 3.74 (2H, s, H-2'') and δ_H_ 3.63 (3H, s) were all observed. The ^13^C-NMR and DEPT spectra showed a total of 41 carbon signals, among which 30 carbons were ascribable to the triterpene aglycone, five carbons to a pentose residue, four carbons to a monomethyl malonate group, and two carbons to an acetyl group (see Table 1). A comparison of the ^1^H- and ^13^C-NMR spectra of **2** with those of actein [13] revealed that **2** has an additional monomethyl malonate unit. Long-range correlations between δ_H_ 4.79 (H-1') and 88.6 (C-3), and between δ_H_ 5.05 (dd, *J *= 3.7, 8.4 Hz, H-12) and 170.6 (12-COCH_3_) were observed in the HMBC spectrum of **2**, which assigned the xylose linked at C-3 and the acetyl connected to C-12, respectively. The xylose was also detected in the aqueous fraction of the acid hydrolysis products of **2**. The additional monomethyl malonate unit was connected at C-2' of xylose, which could be confirmed by HMBC correlation between H-2' (δ_H_ 5.50, t, *J *= 8.4 Hz) and the carbonyl signal at δ_C_ 166.5 (s, C-1'') (see Figure 3). On the basis of the above evidence, the chemical structure of **2** was assigned as 2'-O-(3''-methylmalonyl)-actein, named cimifoside E.

## 3. Experimental

### 3.1. General

Melting points were determined on a Mel-Temp II; Optical rotations were recorded on a Horiba SEPA-300 polarimeter; IR spectra were recorded on a Shimadzu IR-450 instrument, and are reported in cm^−^^1^; UV spectra were obtained in MeOH with a Shimadzu UV-2401A spectrometer, and absorption maxima are given in nm; ^1^H-, ^13^C-, and 2D-NMR spectra (all in C_5_D_5_N) were recorded with Bruker AV400 or DRX500 instruments, using TMS as an internal standard. Mass spectral data were recorded on a VG Autospec 3000 spectrometer. Silica gel (200–300 mesh, Qingdao Marine Chemical, P.R. China), Lichroprep RP-18 (40–63 um, Merck, Darmstadt, Germany) and Sephadex LH-20 (Pharmacia Fine Chemical Co., Ltd.) were used for column chromatography (CC). Fractions were monitored by TLC, and spots were visualized by heating TLC sprayed with 10% H_2_SO_4_.

### 3.2. Plant Material

The rhizomes of *C. foetida* were collected in Lijiang, Yunnan Province, China, in June, 2003 and authenticated by Prof. Zong-Yu Wang (Kunming Institute of Botany, CAS). A voucher specimen (KUN No. 200308025) of the collection has been deposited at State Key Laboratory of Phytochemistry and Plant Resources in West China, Kunming Institute of Botany, the Chinese Academy of Sciences.

### 3.3. Extraction and Isolation

The dried, milled rhizomes of *C. foetida* (23.4 kg) were exhaustively extracted with 90% MeOH (75 L) under reflux (4 times). The extract was evaporated under reduced pressure to yield a syrup-like residue (about 3.9 kg). The syrup was suspended in H_2_O-MeOH (9:1), and extracted successively with EtOAc (15 L, 3 times, room temperature). The organic layer was dried (about 1.7 kg), and then absorbed on silica gel (2 kg), and subjected to column chromatography eluting with a gradient system of CHCl_3_-MeOH from 0 to 100%. The fractions were monitored by TLC, and combined to give seven fractions (Fr.1-7). Fr.2 (55 g), Fr.3 (152 g) contained abundant triterpenoids by TLC examination, indicating the two fractions were worthy of further investigation. Fr. 2 was rechromatographed on a column (silica gel, CHCl_3_-MeOH 100:1, 80:1, 65:1, 50:1) to obtain subfraction Fr.2.1, containing compounds **3** (15 mg) and **4** (79 mg). The subfraction 2.1 was subjected to RP-C_18_ by using MeOH-H_2_O (80:20) as mobile phase to give compounds **1** and **2**. Fr. 3 was subjected to CC (silica gel, CHCl_3_-MeOH 30:1, 20:1, 10:1 gradient ) and further purified by Sephadex LH-20 with eluting MeOH to give **5** (76 mg) and **6** (43 mg).

*Cimiaceroside E* (**1**). white powder; m.p. 172 °C; [α]^ 27^_D_ -25.0° (*c *= 0.80, CHCl_3_: EtOH (1:1)); negative FABMS *m/z* (%) 717 [M-H]^–^ (8), 689 (25), 118 (43), 601 [M-117]^–^ (4) and 117 [C_3_H_7_O_4_]^–^ (44); negative HRFABMS (*m/z *717.3146 [M-H]^–^, calcd. 717.3158 for C_40_H_61_O_11_);IR (KBr) ν_max_ 3454, 2957, 2927, 2870, 1728, 1631, 1442, 1381, 1366, 1339, 1211, 1172, 1072, 1052, 971, 897, 820, 633, 583, 537 cm^−^^1^. ^1^H-NMR (500 MHz, C_5_D_5_N) and ^13^C-NMR data (100 MHz, C_5_D_5_N) see Table 1.

*Cimifoside E* (**2**). white powder; m.p. 168–179 °C; [α]^ 27^_D_ -45.5° (*c *= 1.10, CHCl_3 _: EtOH (1:1)); negative FABMS *m/z* 775 [M-H]^–^, 662 [M-103]^–^ and 104 [C_3_H_4_O_4_]^–^; negative HRFABMS (*m/z *775.4365 [M-H]^−^, calcd. 775.4382 for C_41_H_59_O_14_); IR (KBr) ν_max_ 3455, 2953, 1760, 1737, 1457, 1440, 1369, 1348, 1244, 1204, 1163, 1075, 1042, 1024, 984, 831, 726, 633, 604, 540 cm^–1^. ^1^H-NMR (500MHz, C_5_D_5_N) and ^13^C-NMR data (100 MHz, C_5_D_5_N) see Table 1.

### 3.4. Acid Hydrolysis of Compounds **1**, **2**

Compounds **1** and **2** (6 mg of each) were refluxed with 5% HCl in MeOH (7 mL) for 8 h. Each mixture was diluted with H_2_O and neutralized with NaHCO_3_. The neutral hydrolysate revealed the presence of only xylose by TLC (n-BuOH-AcOH-H_2_O, 4:1:1, R_f_ = 0.4) upon comparison with the authentic sample.

## 4. Conclusions

Due to their medicinal uses, chemical constituents of *Cimicifuga *species have been extensively studied by several groups [7,12,17]. To date, more than 200 triterpenes have been isolated from the genus [4,18]. In our continuing studies, new compounds had still been found. In this paper, new triterpene glycosides with a monomethyl malonate group rarely found in the genus *Cimicifuga* were identified.
